# Stem cell therapy: a new approach to the treatment of refractory depression

**DOI:** 10.1007/s00702-014-1194-2

**Published:** 2014-03-27

**Authors:** Yoshiyasu Kigawa, Eri Hashimoto, Wataru Ukai, Takao Ishii, Kengo Furuse, Hanako Tsujino, Tomohiro Shirasaka, Toshikazu Saito

**Affiliations:** 1 Department of Neuropsychiatry, School of Medicine, Sapporo Medical University, S-1, W-16, Chuo-ku, Sapporo, 0608543 Japan; 2 Department of Psychiatry, Ebetsu City Hospital, Wakakusa-Cho 6, Ebetsu, Hokkaido 0678585 Japan

**Keywords:** Stem cell therapy, Antidepressant, Refractory depression, Fetal alcohol spectrum disorder, Parvalbumin positive interneuron, PSD-95

## Abstract

To better understand the relationship of repeated exposure to adversity during early development as a risk factor for refractory depression, we exposed pregnant female rats to ethanol and the resulting pups to corticosterone during adolescence. A stressful forced swim test was then used to induce depression-like behavior. The adolescent rat brains were examined for the possible therapeutic benefit of a combination of sertraline, an antidepressant, and neural stem cells (NSCs) complexed with atelocollagen in relation to the level of GABAergic interneuron and synaptic protein density in different brain regions. The combined exposures of prenatal and adolescent stress resulted in a reduction in parvalbumin (PV)-positive phenotype of GABAergic interneurons and reduced postsynaptic density protein 95 (PSD-95) levels in the anterior cingulate cortex, amygdala, and hippocampus. Treatments with sertraline and NSCs reversed the reductions in PV-positive cells and PSD-95 levels. Furthermore, the combined treatment of sertraline and NSCs resulted in reduced depressive-like behaviors. These experiments underscore a potentially important role for synaptic remodeling and GABAergic interneuron genesis in the treatment of refractory depression and highlight the therapeutic potential of stem cell and pharmacological combination treatments for refractory depression.

## Introduction

Repeated exposure to environmental adversity during development can cause treatment resistant, refractory depression. Although genetic research has revealed that the vulnerability to depression is partially heritable, there is also strong support for the pathogenic role of early environmental adversity in the development and expression of this disorder. Current antidepressant medications result in an inadequate therapeutic response due to the long delay of a therapeutic effect or a failure of response in many patients (Rush et al. 2006). Patients that are refractory to pharmacological intervention represent a serious clinical problem and improved treatments are needed. Prenatal alcohol exposure has been identified as a potential risk factor for refractory depression and a high rate of co-morbid depression has been reported in fetal alcohol spectrum disorder (FASD) patients (Famy et al. 1998).

Abnormalities in cortical and limbic system development may be linked to refractory depression, but the underlying cellular pathogenesis in the brain remains elusive. The cortex, hippocampus, and amygdala are candidate brain regions since they have also been implicated in the pathophysiology of FASD. These regions have been identified as key structures in memory and emotion (Archibald et al. 2001; Miller 1996). Dysfunction of proper GABAergic inhibition and the consequent imbalance between excitation and inhibition in the corticolimbic neural network underlies at least part of the pathophysiological process of several psychiatric disorders, including FASD, depression, and schizophrenia (Miller 2006; Guidotti et al. 2005). Several studies have shown an alteration in presynaptic and postsynaptic components of the GABAergic systems. A reduction of the rate-limiting synthetic enzyme glutamic acid decarboxylase (GAD) 67 levels has been reported in human brain samples of schizophrenics and animals with FASD (Isayama et al. 2009; Akbarian and Huang 2006). In addition, in brain samples from schizophrenics, parvalbumin (PV)-positive interneurons (many of which display a fast-spiking firing pattern) were severely disrupted (Curley et al. 2011; Gonzalez-Burgos et al. 2010).

Stem cell-based regenerative therapy is considered a promising cellular therapeutic approach for some patients with incurable brain diseases. Some studies have shown that stem cell treatments improve behavioral deficits in animal models of neurological disorders such as Parkinson’s, Huntington’s, and Alzheimer’s diseases. Although many ethical and technical issues still need to be resolved, this approach could possibly be useful for treating affective disorders and schizophrenia after other available medications have been unsuccessful.

Previously, we found that intravenous neural stem cell (NSC) treatment reversed the reductions in the number of GABAergic interneurons and synaptic protein density in the brain decreased by prenatal ethanol exposure. Further, treatment using NSCs also reversed impaired memory/cognitive function and social interaction behaviors in rats tested using a fetal environmental stress-induced psychiatric disorder model (Shirasaka et al. 2012). Whether this approach would be successful in treating the refractory type of depression caused by the combined exposure to stress during the fetal and adolescent period of development has not yet been tested.

To test this question, we studied the involvement of corticolimbic GABAergic interneuron disruption in a rat model of refractory depression and followed by stem cell treatment. As a refractory depression model, we first exposed the animals to ethanol prenatally and then to corticosterone (CORT) during the adolescent period. In a clinical point of view, “refractory depression” usually includes treatment-resistant depression. Although we need precise studies to define it as a treatment-resistant refractory depression model, we produced a novel psychiatric animal model for disturbed neurodevelopmental aspects by combining with fetal alcohol exposure and corticosterone-mediated early life stress, as one kind of refractory depression model. We show here that aspects of depressive-like behavior resulting from a forced swim test (FST) are reversed by the combined treatment of an antidepressant and the intravenous administration of fetal rat brain-derived NSCs with atelocollagen to reduce immune rejection and to potentiate effective migration of administrated cells into the brain. In addition, we show that alterations of PV-containing GABAergic interneurons and synaptic density protein levels result from the combined treatment of antidepressant drug and NSC cells in this model. Our results establish a new role for PV-containing GABAergic interneurons associated with treatment resistant, refractory depression-like behavior in rats and suggest possible new therapeutic options for stem cell therapies for treating refractory types of psychiatric disorders.

## Methods

### Materials

The following materials were purchased: Hank’s balanced salt solution, neurobasal medium, B27 and 5-(and-6)-carboxyfluorescein diacetate succinimidyl ester (CFSE) from Invitrogen (Carlsbad, CA, USA); recombinant human fibroblast growth factor-2 from Pepro Tech (London, UK); atelocollagen from Koken (Tokyo, Japan); CORT and sertraline HCl from Tokyo Chemical Industry (Tokyo, Japan).

### Experimental design and prenatal ethanol treatment

**Fig. 1:**
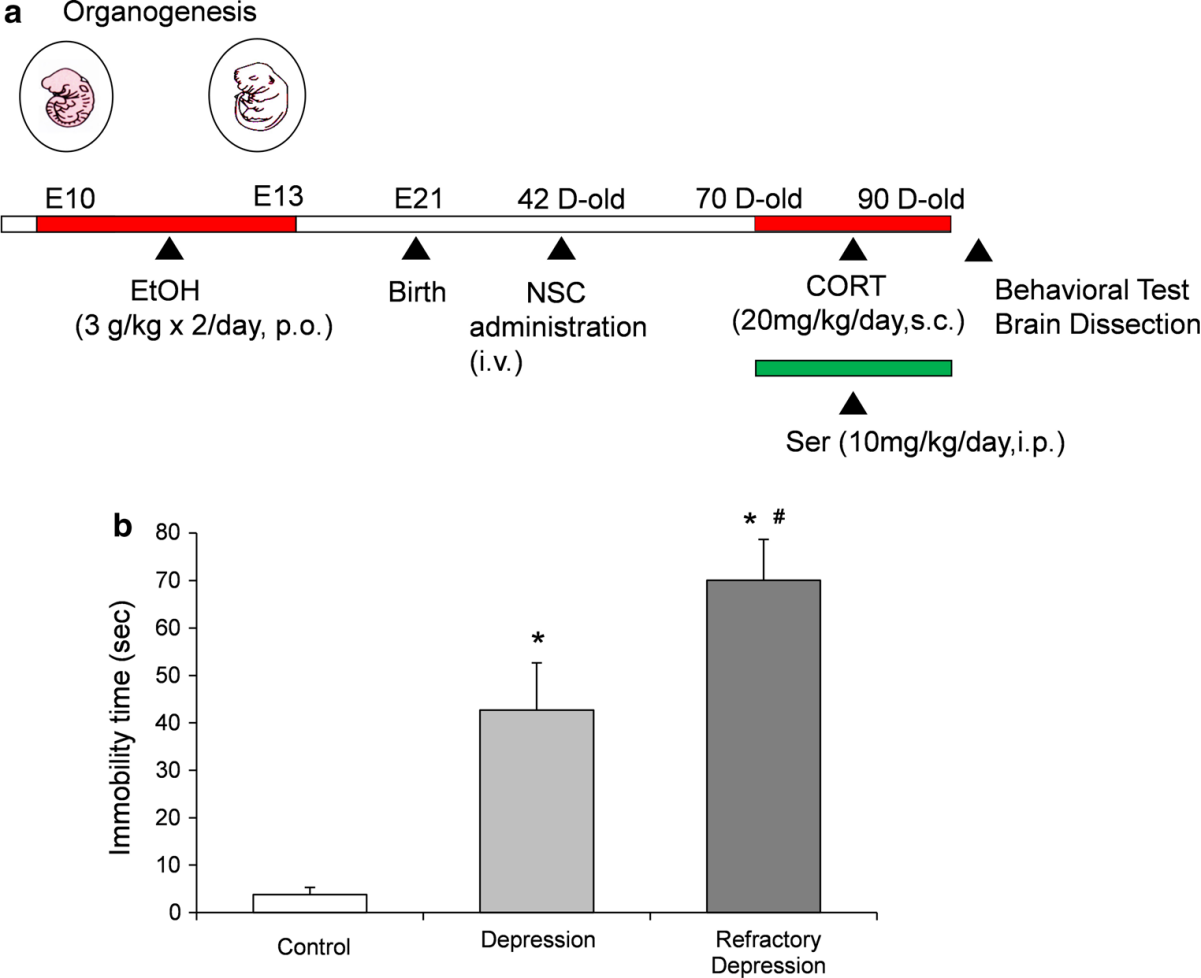
Experimental design and behavioral test. **a** Experimental schedule. Fetal period stress was obtained by oral treatment of ethanol (3 g/kg × 2/day) during the pregnancy, from embryonic days (*E*) 10–13. CSFE fluorescent-labeled NSC solution mixed with atelocollagen was injected intravenously into 42-day-old rats. CORT (20 mg/kg/day s.c.) and/or Ser (10 mg/kg/day i.p.) were administered repeatedly every day for 21 days (70–90 days old). Alterations of behavioral performance and brain neural circuit were evaluated 91 days old. On 90 days old, rats were forced to swim for a 15-min period, subsequently on 91-day-old rats were subjected to the second 5-min forced swim test session, and the total duration of immobility was measured by video camera recordings. **b** Changes of immobility time in forced swim test. Immobility times in depression model rats (CORT) were significantly prolonged than control rats (*P < 0.05). Refractory depression model rats (prenatal ethanol exposure + CORT) displayed significantly longer immobility time than control rats (*P < 0.05), and depression model rats (^#^ *P* < 0.05). Data represent mean ± s.e.m. (*n* = 10–13)

Pregnant Wistar rats were purchased from Clea Japan (Sapporo, Japan). Rats were administered ethanol (3 g/kg po twice a day; estimated mean whole blood concentration 150 mg/dl) or an equivalent volume of physiological saline via an intragastric catheter every 12 h for 4 days on gestational days 10–13 as described previously (Yoshinaga et al. 2007). Each rat was housed individually and was allowed to give birth. The pups were fed by their biological lactating mothers and weaned at 30 days. Rats were housed at 22 °C on a 12:12-h light–dark cycle with ad libitum fed. All experimental procedures were approved by the institutional animal care committee and conducted following the Sapporo Medical University Guidelines for the Care and Use of Laboratory Animals. The experimental design is shown in Fig. 1a.

### Sertraline (Ser) and Corticosterone (CORT) administration

Ser was dissolved in physiological saline and injected 10 mg/kg ip CORT was suspended in physiological saline containing 1 % dimethylsulfoxide (DMSO) and 1 % Tween80 and injected 20 mg/kg sc (Zhao et al. 2008). All treatment procedures were completed during the light phase, once a day every day for 21 days. The groups consisted of: (1) Ser and CORT; (2) vehicle and CORT; and (3) vehicle and vehicle.

### Forced swim test (FST)

FST is a behavioral test used frequently to evaluate the efficacy of potential antidepressant drugs in rat and mouse models. The immersion of rats in water for an extended period of time produces a characteristic behavior of immobility (cf., learned helplessness) (Detke et al. 1995). The administration of a antidepressant medication, such as sertraline, typically decreases the immobility behavior. A transparent plexiglas cylinder (50 cm high × 20 cm wide) was filled to a depth of 30 cm with water at 25 (±1) °C. Two swim sessions were conducted: an initial 15-min pretest followed 24 h later by a 5-min test. Immobility behavior was calculated as the length of time in which the animal did not show escape responses. Following both swim sessions, the rats were removed from the tank, dried with towels and placed in heated cages, and then returned to their home cages. Test sessions were recorded with a videos camera and mounted 1 m directly above the tank. The water in the swim tank was changed between animals. All rats were exposed to the FST 24 h after their final CORT injection.

### NSC preparation and labeling

NSCs were obtained from 13.5-day-old rat embryos and cultured in a monolayer as (Tateno et al. 2005). Briefly, telecephalic neuroepithelium was dissected and trimmed in ice-cold Hank’s balanced salt solution. Cells were dissociated by mechanical trituration and collected by centrifugation (300 g for 5 min at 4 °C). The dissociated cells were planted in culture dishes coated with poly-l-ornithine/fibronectin in neurobasal medium supplemented with 2 % B27, 0.5 mM l-glutamine, and 20 ng/ml fibroblast growth factor-2 at a density of 5 × 10^4^ cells/cm^2^. After 7 days of cell expansion culture under 5 % CO_2_ at 37 °C, cells were stained with fluorescein-based dye to trace their migration by incubating the cells in phosphate-buffered saline buffer containing 5 μM CFSE in constant agitation for 15 min at 37 °C before administration. The dye (highly fluorescent, amine-reactive carboxyfluorescein diacetate succinimidyl ester; CFSE) and protein (intracellular amines) adducts that form in labeled cells are retained by the cells throughout development, meiosis, and in vivo tracing (Wang et al. 2009).

### NSC administration procedures

Although stem cell treatment has potential benefits for severe and currently incurable neuropsychiatric disorders, certain risks may result including tumor formation, immune rejection of administered stem cells, hemorrhage during neurosurgery, and postoperative infection. To minimize invasiveness and the risk of tumor formation by NSC treatment, we chose intravenous administration. The level of tumor risk depends upon the degree of histocompatibility of the cells and other factors (cell number, injection site, disease model, etc.). A very low risk of teratomas has been reported using intravenous NSC administration (Dressel 2011). In our previous work, it was indicated that intravenously injected NSCs were detected in the brain by visualizing fluorescent marker (CFSE), and the number of injected NSCs in brains of fetal alcohol effects model rats was greater than in the control rat brains in all four areas [cortex, hippocampus, striatum, and subventricular zone (SVZ)], as demonstrated by RI ([^35^S]-methionine) labeling (Yoshinaga et al. 2007). In this study, at 42 days old, a NSC suspension (5 × 10^6^) mixed with 0.02 % atelocollagen dissolved in saline in 0.5 ml total fluid volume was injected slowly into the rat’s tail vein over 1 min. We have established that 0.02 % of atelocollagen does not influence any NSC functions, including proliferation, migration, differentiation, and survival. Further investigation is needed in an in vivo study to determine the effects of intravenous injection of atelocollagen alone (Yoshinaga et al. 2013).

### Western blot

After the FST, all animals were deeply anesthetized with isoflurane and killed. Immediately thereafter, blood was collected and crude dissection of the brains was conducted; the anterior cingulate cortex and amygdala tissues were punched out and cryopreserved in −80 °C. Subsequently, tissue was thawed and total protein was produced using RIPA Lysis Buffer (Santa Cruz). Protein concentration was determined using a BCA kit (Thermo Scientific, Rockford, IL, USA). Serum BDNF concentrations were measured and protein samples underwent western blot analysis as described previously. Briefly, 20 μg aliquots were subjected to sodium dodecyl sulfate–polyacrylamide gel electrophoresis on 10 % polyacrylamide gels and transferred to polyvinylidene difluoride membranes. After blocking with PVDF Blocking Reagent (TOYOBO, Osaka, Japan) overnight at 4 °C, blots were probed with anti-postsynaptic density protein 95 (PSD-95) (rabbit, 1:1,000; Cell Signaling Technology, Danvers, MA, USA) and glyceraldehyde 3-phosphate dehydrogenase (rabbit, 1:200; Santa Cruz) for 1.5 h at room temperature, then washed and incubated for 1 h with horse radish peroxidase-conjugated anti-rabbit immunoglobulin G (1:2,000; Dako Cytomation, Glostrup, Denmark), respectively. Immunoreactive bands were detected with an enhanced chemiluminescence system (ECL system; GE Healthcare, Waukesha, WI, USA), and densitometric data were quantitatively analyzed by capturing images using a Sensobation Camera (UVP, Upland, CA, USA) in conjugation Vision Works LS ver.6.1.1 (UVP) software.

### Immunohistochemistry and PV-positive cell counts

After completing behavioral testing, rats were deeply anesthetized with isoflurane and transcardially perfused with heparinized saline (0.5 %) followed by 4 % paraformaldehyde in phosphate buffer. The brains were then immersed in 4 % paraformaldehyde, paraffin embedded and cut serially at the coronal plane into 20 μm sections. A portion of the intravenous administered NSCs that migrated into the brain were identified as GABAergic interneurons by double imaging of fluorescent CFSE dye labeling and immunostaining of GAD67 in the same visual field in the anterior cingulate cortex, hippocampus, and amygdala. Briefly, sections were immunostained for GAD67 (rabbit anti-GAD67, 1:30; Santa Cruz, CA, USA) detected with Avidin and Biotinylated Complex (ABC) system (45 min, Vectastain ABC kit, Elite PK-6100, Vector Laboratories) and visualized with 3,3′-diaminobenzidine (SK-4100, Vector Laboratories, Burlingame, CA, USA), followed by the detection of double-labeled cells with fluorescent dye and 3,3′-diaminobenzidine. To obtain accurate counts of PV-positive interneurons, we performed immunohistochemistry using an antibody for PV (mouse anti-PV, 1:2,000; Sigma, St Louis, MO, USA) detected with the ABC system visualized with 3,3′-diaminobenzidine. Three pairs of animals in each group (control Refractory Depression; Refractory Depression + Ser; Refractory Depression + NSCs; and Refractory Depression + NSCs + Ser) were examined. PV-positive cells were counted within a standardized rectangular area of 0.574 mm^2^ in the anterior cingulate cortex and amygdala, and 3.533 mm^2^ in hippocampus. Counts were performed in at least six slices selected at the same level for each animal. Sections were mounted with fluorescent mounting solution (Dako, Glostrup, Denmark), covered with a cover slide and sealed. Digital images were obtained with a fluorescence or optical instrument, Olympus BX52TF microscope (Olympus, Tokyo, Japan).

### Statistical analysis

One-way analyses of variance (ANOVA) followed by Tukey’s HSD post hoc comparison were used to determine statistical significance. *P* values were set at *P* < 0.05 for all experimental observations tested by ANOVA. All values included in the figure legends represent the mean ± sem. Statistical analyses were performed using SPSS 11.0 for Windows (SPSS Japan, Tokyo, Japan).

## Results

### Establishing an animal model of refractory depression by exposure to early developmental adversity

We established a refractory depression rat model using alcohol exposure during the fetal period followed by chronic CORT treatment during adolescence. Three months after administration of NSCs and adolescent CORT treatment, rats were tested for their depressive-like behavior by measuring their immobility time in a FST (Fig. 1a).

Chronic CORT-induced depressive model rats displayed 10 times longer immobility time (immobility time 43 ± 10.0 s/5 min) than the control rats (immobility time 4 ± 1.6 s/5 min). The fetal alcohol + adolescent CORT-induced refractory depression model rats showed significantly longer (~1.6-fold) immobility time (immobility time 70 ± 8.6 s/5 min) than CORT alone induced depressive model rats, demonstrating the severity of the depressive-like behavior in the refractory depression model rats (Fig. 1b).

### Effects of sertraline, NSC administration, and their combined effects on refractory depression

**Fig. 2:**
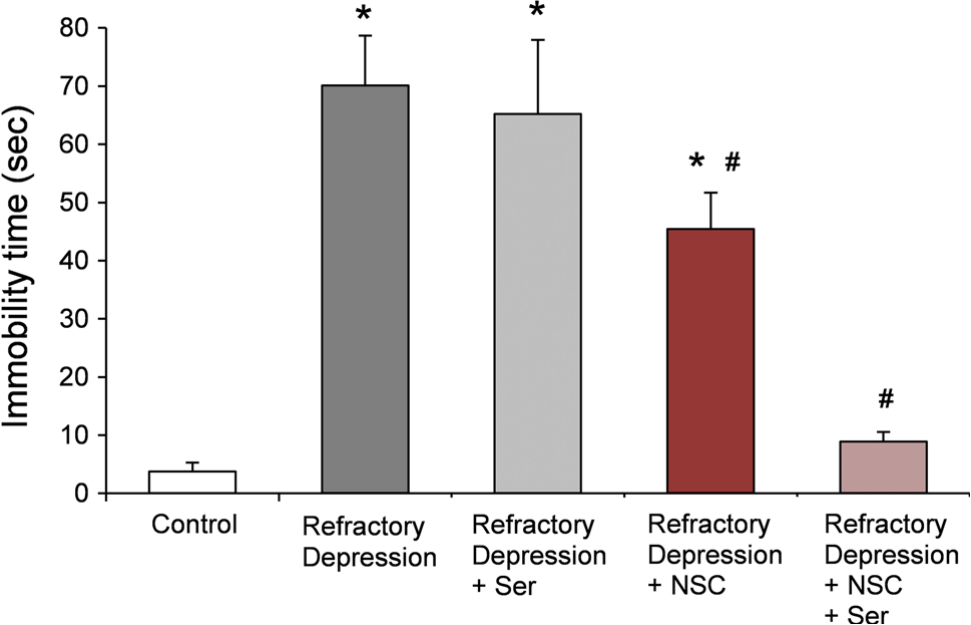
Effects of three types of treatment on refractory depression model rats. Refractory depression model rats (prenatal ethanol exposure + CORT) displayed significantly longer immobility time than control rats (*P < 0.05). NSCs and NSCs + Ser treated rats significantly shortened their immobility times compared to refractory depression model rats. Rats treated with Ser alone did not show any change in immobility time comparing to refractory depression model rats (*P < 0.05 compared to control, ^#^ *P* < 0.05 compared to refractory depression model). Data represent mean ± s.e.m. (*^, #^ *P* < 0.05)

To examine the effects of sertraline, NSCs, and the combined treatment of drug + NSCs on depressive-like behavior in a refractory depression rat model, we treated animals with an antidepressant [sertraline (Ser) 10 mg/kg/day, 21 days, ip], NSCs (a single iv injection of 5 × 10^6^ cells), or combination of Ser and NSCs, and evaluated the potential influence of these treatments on severe depressive-like behavior following a FST. There was no significant change in the immobility time between fetal alcohol + adolescent CORT-induced refractory depression model rats and the refractory depression model rats with Ser treatment (immobility times; refractory depression model, 70 ± 8.6, Ser treatment, 65 ± 12.8 s/5 min). However, the addition of NSC treatment significantly reduced the immobility time of refractory depression model rats (immobility time 45 ± 6.3 s/5 min). Thus, rats receiving the combined treatments of Ser and NSCs displayed immobility times approximately one-seventh of the refractory depression model rats (immobility time 9 ± 1.7 s/5 min) and similar that of control rats, suggesting a potential synergistic effect of this combined treatment of drug + NSC cells (Fig. 2).

### Detection of administered NSCs in brain regions

Although we could not confirm complete absence of tumorigenic stem cells histochemically, the intravenous administration of NSCs to refractory depression model rats did not produce any teratoma formation or growth throughout the experiments.

**Fig. 3:**
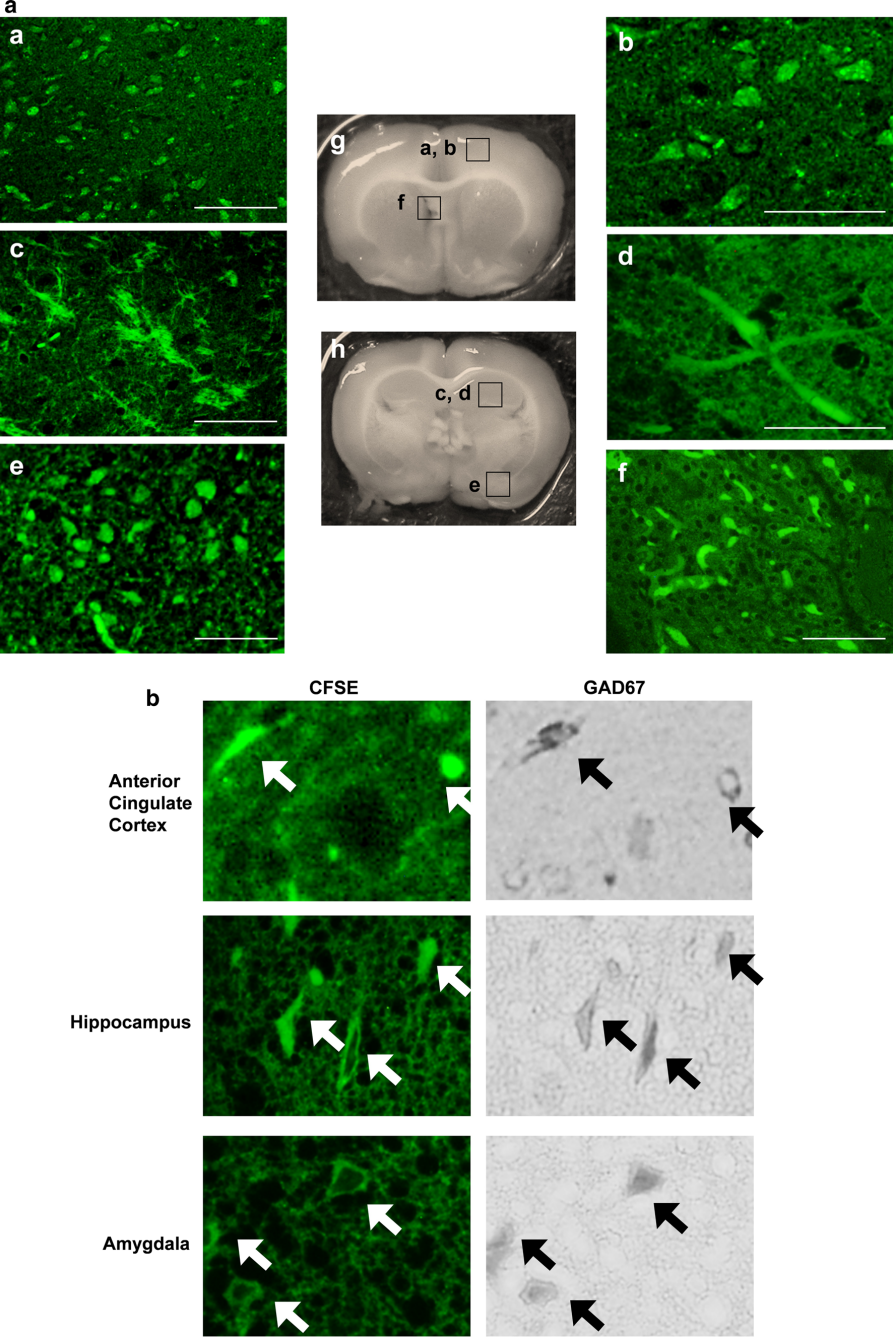
**a** Identification of migrated NSCs in brain. *a* Intravenously administered CSFE-labeled NSCs widely detected in brain involving *a*, *b* anterior cingulate cortex, *c*, *d* hippocampus, *e* amygdala, *f* choroid plexus, and by fluorescent images. *g* Coronal sections of anterior side (Bregma A 1.00 mm), and *h* posterior side (Bregma P2.20). *Scale bars* 50 μm. **b** Identification of administered NSCs as GABAergic cells. Fluorescent images show administered cells (CSFE in *green*) in anterior cingulate cortex, hippocampus, and amygdala. Same cells were immunostained with GABAergic neuron marker (GAD67 in *gray*) in light photomicroscopic images

The ease of treatment and clinical precedent (Sasaki et al. 2011; Mitrecic 2011) renders intravenous administration desirable. However, several studies have reported poor cell delivery to the brain due to the blood–brain barrier and suggest cell entrapment in peripheral organs (Gao et al. 2001). To reduce immune rejection and to increase the entry of administrated NSCs into brain, we pretreated NSCs with atelocollagen, a recently developed means of promoting gene delivery. This pre-treatment achieved an approximate twofold higher migrating ratio of NSCs into brain in response to intravenous administration of NSCs in FASD model rats (Yoshinaga et al. 2013). Prenatal ethanol exposure specifically reduces the density of the cortical GABAergic population in rodents (Bailey et al. 2004) and reductions in GABA activity have been reported in adolescents with depression (Sanocora et al. 1999). Therefore, to achieve regeneration of GABAergic neuronal circuits which are assumed to be impaired in refractory depression model rats, we prepared fetal rat telencephalon-derived NSCs for treatment by monolayer cultures, a method which generates GABAergic neurons efficiently (Shin et al. 2012). To determine the potential migration of NCSs into different brain regions, we labeled NSCs with CFSE, a fluorescent staining dye that can label all NSCs regardless of their differentiation (Wang et al. 2009). We were able to detect CFSE-positive cells in several areas of refractory depression rat brain at 3–4 months post-treatment (Fig. 3a). These marked cells also distributed in control rat brain (data not shown). In fluorescence detection, we used Olympus IX71 inverted system microscope combined with the new UIS2 optical system (Olympus, Tokyo, Japan). In the UIS2 system, fluorescence S/N ratio is improved using selected low fluorescence glass material, and minimized autofluorescence obtained by anti-reflection coating and cementing material. In addition, to minimize an autofluorescence caused by the hemoglobin and derivatives of hemoglobin containing in red blood cells in a brain tissue, we transcardially perfused heparinized saline and paraformaldehyde in phosphate buffer carefully as described in methods. A high proportion of the injected were expected to differentiate into GABAergic neuron phenotypes due to the culture method used in this study. Indeed, a portion of the administered cells were identified as GAD67-containing cells in the anterior cingulated cortex, amygdale, and hippocampus (Fig. 3b). GAD67 has been reported specifically reduced in cortical PV interneuron axon terminals in some psychiatric disorders (Curley et al. 2011). We previously demonstrated that intravenously administered [^35^S]-methionine labeled NSCs survived and distributed throughout FASD rat pup brains at 4 months after treatment. Further, greater radioactivity was noted in the cortex, hippocampus, striatum, and SVZ regions in FASD rat brains compared to controls (Yoshinaga et al. 2007). The distribution difference between the control and refractory depression brains needs to be more precisely examined using more sensitive techniques, such as flow cytometry. The current study’s findings indicate that intravenously administered NSCs could migrate across the blood brain barrier and delivered to the cortical and limbic brain areas with a survival of at least 4 months. The molecular mechanisms by which NSCs cross the blood brain barrier are uncertain but some hypotheses exist, including the implications of a direct effect of NSCs on blood brain properties (Lim et al. 2007) and microchimerism of fetal brain-derived NSCs (Dawe et al. 2007).

### Alteration of PV-positive interneuron densities

**Fig. 4:**
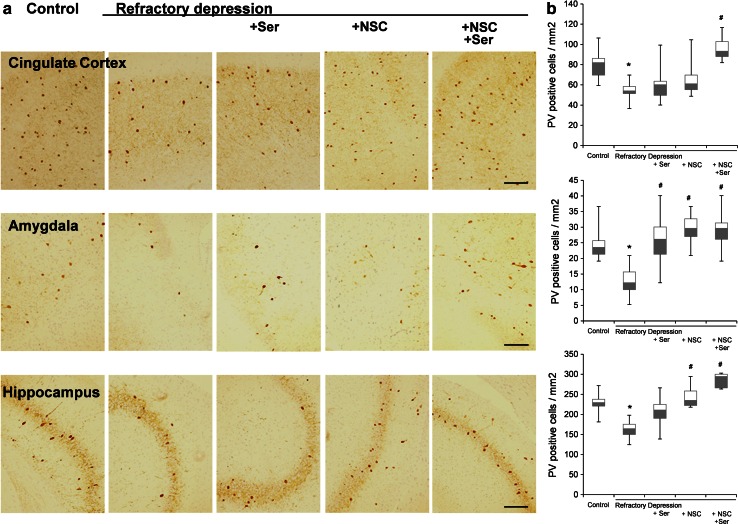
Decreased number of PV-positive interneurons and their area-dependent reverses by various treatments. **a** PV-positive cells were counted in coronal sections from control, refractory depression, refractory depression + Ser, refractory depression + NSCs, and refractory depression + NSCs + Ser treated rats. **b** Immunohistochemistry with DAB-labeled anti-PV antibody indicated that the amount of PV-positive cells significantly decreased in anterior cingulate cortex, amygdala, and hippocampus in refractory depression model rats (*P < 0.05). In cingulate cortex, combined treatment of NSCs and Ser significantly reversed the reduction of these cells. In amygdala, all three treatments of Ser, NSCs, and NSCs + Ser significantly recovered these reductions. In hippocampus, both treatments of NSCs and combined treatment of NSCs and Ser significantly recovered these reductions. (^#^ *P* < 0.05). Data represent mean ± s.e.m. (*n* = 3–4, two sections per rat). *Scale bars* 100 μm

We examined whether GABAergic interneurons, especially PV-positive cells known to correlate with cognition and executive dysfunction in schizophrenia (Nakazawa et al. 2012) and depressive-like behavior and the antidepressant effects in depression (Pozzi et al. 2014; Zhou et al. 2012) are altered in a refractory depression rat model and NSC treatment. We discovered that, in refractory depression model rats, the number of PV-positive interneuron was decreased in the anterior cingulated cortex, amygdala, and hippocampus. Further, the combined treatment of an antidepressant, sertraline, and NSCs significantly attenuated these reductions in all three brain regions (Fig. 4a, b). In contrast, treatment with NSCs alone significantly ameliorated these reductions in the amygdala and hippocampus, but not in the cingulate cortex. Treatment with sertraline alone reversed the reduction only in the hippocampus (Fig. 4a, b). These results implicate the upregulation of PV-positive interneurons, especially in the anterior cingulate cortex, in the recovery effects of NSCs and sertraline combination on depressive-like behavior.

### Alterations of PSD-95 protein levels

**Fig. 5:**
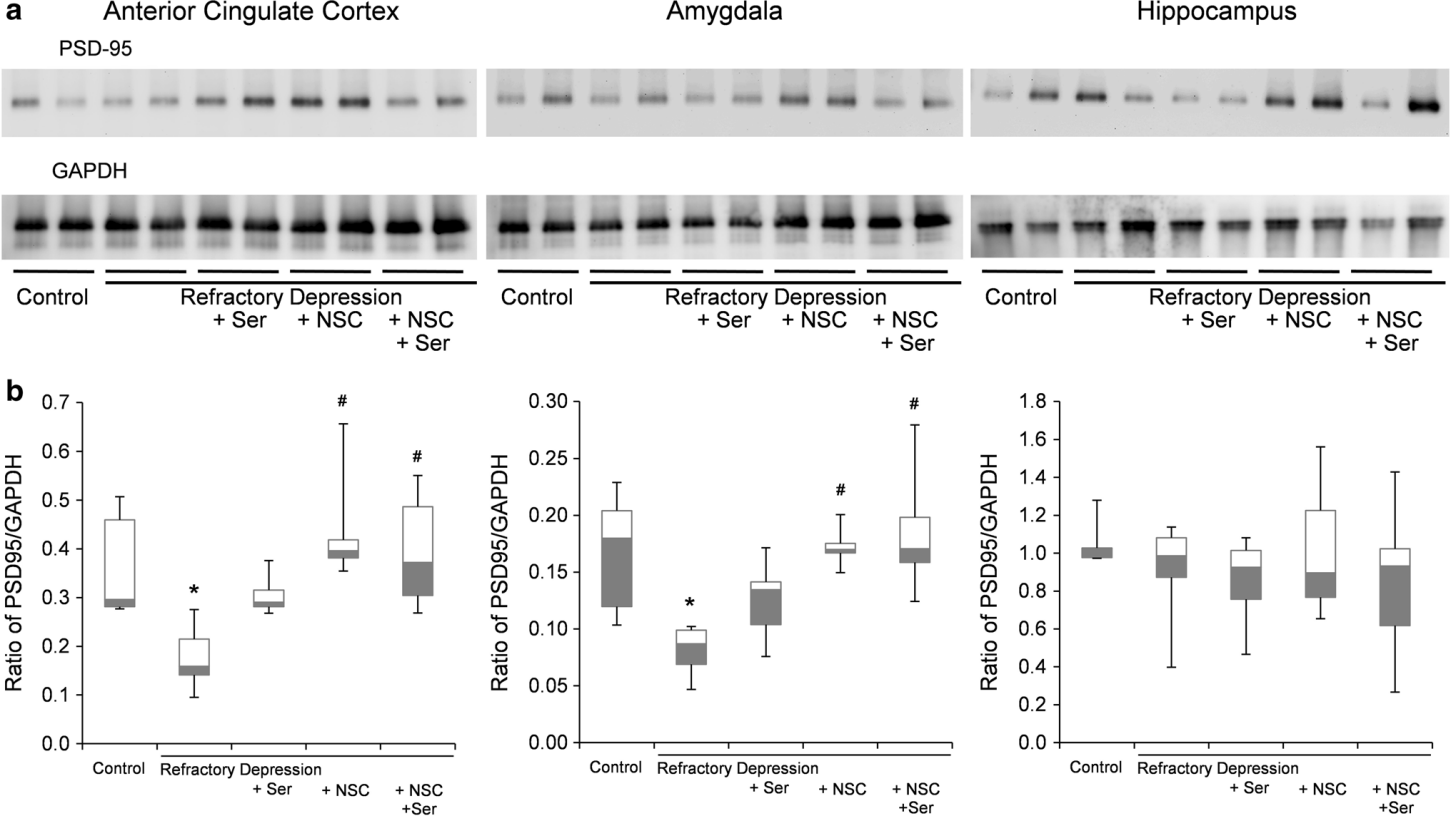
Decreased synaptogenesis in refractory depression model rats and their reverse by various treatments. **a** Amount of synapses was represented by PSD-95 protein levels in western blot analysis. **b** Values of PSD-95 protein levels normalized to GAPDH were calculated in lysates from control, refractory depression model, refractory depression + Ser, refractory depression + NSCs, and refractory depression + NSCs + Ser rats. PSD-95 protein levels significantly decreased in anterior cingulate cortex and amygdala in refractory depression rats (*P < 0.05 compared to control). Whereas, NSCs and NSCs + Ser treatments reversed these reductions in both areas (*P < 0.05 compared to control, ^#^ *P* < 0.05 compared to refractory depression). In contrast, in hippocampus, PSD-95 protein levels did not change in all groups. Data represent mean ± s.e.m. (*n* = 4–6)

To investigate the mechanisms underlying the lasting changes in GABAergic interneuron density, particularly PV-positive cell survival/generation, we hypothesized that synaptic remodeling may be associated with the pathophysiology of refractory depression and the activity resulting from NSC treatment. We measured protein expression levels of PSD-95 in the anterior cingulate cortex, amygdala, and hippocampus of control and refractory depression model rats, with and without subsequent treatment with sertraline or NSCs or their combination. We observed significant changes of protein expression in the anterior cingulate cortex and amygdala. PSD-95 protein levels were decreased in refractory depression model rats; NCSs and the combined treatment of NSCs and Ser reversed the protein expression reductions in these regions. In contrast, Ser treatment alone did not show any significant reductions (Fig. 5a, b).

## Discussion

As a current method to examine restoration of functional circuits in laboratory models of neurodegenerative pathologies, intracerebral injection of NSCs is most commonly used (Master et al. 2007; Dressel 2011). Although this method can produce direct recovery of damaged neural circuits, intraparenchymal injections into induced brain lesions is not an appropriate way to address diffuse or undefined cerebral pathologies in psychiatric illness (e.g., depression, schizophrenia and developmental disorders). The recent introduction of intravenous NSC administration overcomes this limitation in that NSC cells can reach the cerebral parenchyma and induce recovery in models of multiple sclerosis (Barzilay et al. 2011), Huntington’s disease (Lee et al. 2005), and stroke (Honmou et al. 2011). Another advantage is that intravenous NSCs can reduce the risk of tumor formation, invasion, and social debate regarding new trials of regenerative medicine in psychiatric disorders. Nevertheless, medical treatments using fetal brain-derived NSCs or embryonic stem cells raise ethical dilemmas (Master et al. 2007). Further work is needed to examine the efficiency when harvesting recent potential cell sources, such as mesenchymal stem cells (Suzuki et al. 2013), induced pluripotent stem cells (Tsuji et al. 2011), and stimulus-triggered acquisition of pluripotency cells (Obokata et al. 2014).

Using the current method, we found that a specific behavioral abnormality, immobiolity, was potentially reversible in refractory depression model rats induced by both a fetal (ethanol) and adolescent (corticosterone) stressor. This resulted in neural circuit modifications in the anterior cingulate cortex, amygdala, and hippocampus at the level of specific GABAergic interneuron density and changes in synaptogenesis. We discovered that depressive-like behavior, as induced by a FST, is more severe in prenatally ethanol + adolescent CORT-exposed rats than in CORT alone exposed rats. Further, immobility was significantly attenuated by the combined treatment of intravenous administration of NSCs at a time point when neural circuit development is already completed and antidepressant was administered during adolescent stress exposure. In addition, in the amygdala, each treatment of sertraline, NSCs, and their combination restored the reduction of PV-positive interneurons. However, in the hippocampus antidepressant treatment alone did not recover these cell reductions. Furthermore, in the cingulate cortex, the combined treatment of NSCs and sertraline, but not the other treatments did not recover the decrease in these cells. In anterior cingulate cortex and amygdala, the treatment with NSCs and NSCs + sertraline, but not sertraline alone, also recovered the decrease in protein levels of PSD-95 and increased PSD-95 in amygdala. Although parallel findings between PV-positive cell density and PSD-95 expression changes were not found in these three regions, the present findings could have profound implications for the treatment of a kind of refractory, treatment-resistant depression (Samuels et al. 2011), which was produced by combination of prenatal alcohol exposure and adolescent CORT treatment. Antidepressant medication is often ineffective and seldom ‘cures’ the disease. This underscores the necessity for a better understanding of the pathological features in the brain affecting refractory depression and the need for better therapeutic methods to modify their functioning.

The embryonic GABAergic system is vulnerable to various stressors (Cuzon et al. 2008), and prenatal ethanol exposure can cause gross morphological changes in GABAergic cells during development. Here, we found that the number of PV-positive interneurons was reduced in the anterior cingulate cortex, amygdala, and hippocampus of refractory depression model rats. Since PV-positive cortical interneurons seem to be especially vulnerable to this type of developmental stress and the associated disruption of these cells has been reported in various mental disorders, such as schizophrenia and depression, these findings indicate that loss of cortical and limbic PV-positive interneurons might also play a pivotal role in the pathophysiology of refractory type of depression. In addition, we observed that levels of PSD-95 were decreased in the anterior cingulate cortex and amygdala in this model. Recent findings suggest a new role for PSD-95 in excitatory synapse development through a stabilizing effect by neuregulin 1 and its receptor ErbB4, which is specific to PV-positive GABAergic interneurons (Ting et al. 2011). Therefore, our data showing PSD-95 level changes in anterior cingulate cortex and amygdala may be related to the results of PV-positive cell reductions in these areas. Impaired function in recognition and memory related to abnormal synchronized oscillatory activity of principal cortical neurons has been found in schizophrenia. The ability of cortical PV-positive fast-spiking interneurons to drive synchronous oscillatory activity at gamma frequency is recognized as the cellular basis for cognitive and executive brain function (Sasaki et al. 2011). Although the role of PV-positive cells in the pathophysiology of depression is not completely known, it is plausible that a reduction of PV-positive interneurons in the anterior cingulate cortex in refractory depressed model rats could correlate with the impaired mood related behavior produced by the FST. Further research is needed to understand the mechanisms of depressive-like behavior from this refractory depression model, especially the implications for other subpopulations of GABAergic interneurons including somatostatin- and NPY-positive neurons.

PV-positive interneuron reductions and PSD-95 decreases were most evident in the amygdala in the refractory depression model compared to controls. Although studies of GABAergic interneuron dysfunction in amygdala are inconclusive at this point, many researchers have suggested that disruption of amygdala function results in disturbance of the function mood stabilization. For example, since PV-positive interneuron loss in lateral amygdala has been suggested in maternal deprivation stress exposed rats (Mitrecic 2011), our results showing PV-positive interneuron loss in amygdala may represent an important neural circuit disruption related to the pathophysiology of refractory depression.

There are several notable findings of the current study. In this study, the combined treatment of sertraline and intravenous NSCs administration enveloped with atelocollagen restored reductions in PV-positive interneurons in the anterior cingulated cortex, amygdala, and hippocampus. This was associated with the recovery of reduced PSD-95 levels in the refractory depression rat model. Furthermore, combined NSC treatment utilizing atelocollagen plus sertraline displayed potential therapeutic efficacy against depressive-like behavior. Although, the mechanism underlying the effects of NSC + drug treatment cannot be easily explained, there may be several possibilities accounting for their beneficial actions. One is a neuroprotective effect exerted by the administered NSCs. The administration of olfactory ensheathing cells into the dorsal hemisected spinal cord has recently been reported as producing improved functional outcome and elevation in BDNF levels (Gao et al. 2001). Another possibility is the upregulation of neurogenesis. New generation and migration from precursors in SVZ or NG2+ cells within the cortex may be facilitated by the trophic effect of administered NSC and also by antidepressants (Wang et al. 2009; Dayer et al. 2005; Ohira et al. 2013). Another possible mechanism is that administered NSCs integrate into tissue and replace damaged cells. Our immunohistochemical data suggest that a portion of intravenously administered CFSE-labeled NSCs were found in the anterior cingulate cortex, hippocampus, and amygdala, including the area where the PV-positive neuron number reversal occurred following treatment. Some of the CFSE-labeled NSCs showed GAD67 positivity, the protein known to be involved in the axon terminals of PV interneurons (Curley et al. 2011). In a previous study, we have investigated the peripherally injected cell migration and distribution in the brain using double labeling with CFSE and [^35^S]-methionine. This method does permit quantitative analysis (Yoshinaga et al. 2007). However, due to our desire to use neurohistochemical and molecular level analysis, we chose to label the cells only with CFSE dye in this study. Additional investigation is needed to clearly characterize the full potential of administered NSCs to differentiate into GABAergic interneurons and their possible contribution to depression-related behavioral recovery.

In the hippocampus, prenatal ethanol and adolescent CORT exposure did not induce change of the levels of PSD-95. It is known that the effect of prenatal ethanol depends upon the stage of the development at the time of exposure. A recent report demonstrated that TUNEL analysis of E11–E14 ethanol treated embryos (similar to our protocol: E10–E13) at E14 showed a significant increase in apoptotic cells in the cortex, but not in the hippocampus. This suggests a region specific or a developmentally related cell death effect. The hippocampal PSD-95 protein level change was apparently different from those in other areas. PSD-95 was not changed by prenatal ethanol exposure and adolescent stress exposure or any treatment tested here. PSD-95 is a synaptic protein contained in the glutamatergic neurons, therefore, the data may show the predominant state of excitatory cells in the hippocampus caused by ethanol treatment (Kim et al. 2010).

We view the PV-positive interneuron as a prototypical example of how combined stress during of fetal and adolescent period exposures induce refractory depression and NSC treatment produces neural circuit and behavioral changes. Many questions remain about the mechanisms by which refractory depression disorder and NSC treatment cause changes in the GABAergic neuron system. However, the results of the present study provide fundamental new information concerning the molecular/cellular mechanisms underlying the deleterious effects of fetal and adolescent period stress exposures on the brain and their potential reversal by intravenous treatment with NSCs, especially when combined with a medication. More generally, our results provide further support for the notion that intravenous treatment of NSCs complexed with atelocollagen has therapeutic potential for the pharmaceutical intervention of treatment-resistant psychiatric disorders.
